# Animal Models of Diabetes and Related Metabolic Diseases

**DOI:** 10.1155/2019/6147321

**Published:** 2019-01-06

**Authors:** Tomohiko Sasase, Fatchiyah Fatchiyah, Katsuhiro Miyajima, Masayo Koide

**Affiliations:** ^1^Biological/Pharmacological Research Laboratories, Central Pharmaceutical Research Institute, Japan Tobacco Inc., Osaka, Japan; ^2^Department of Biology, Faculty of Mathematics and Natural Sciences, Research Center of Smart Molecule of Natural Genetics Resources, Brawijaya University, Malang, East Java, Indonesia; ^3^Department of Nutritional Science and Food Safety, Faculty of Applied Bioscience, Tokyo University of Agriculture, Tokyo, Japan; ^4^Department of Pharmacology, University of Vermont College of Medicine, Burlington, VT, USA

Metabolic syndrome including diabetes and its complications (e.g., obesity, dyslipidemia, and hypertension) are common diseases and frequently occur in combination. The animal models of metabolic diseases are essential tools to reveal the pathophysiology and provide novel insights to develop new therapies and drugs. To this day, many animal models such as hereditary models, chemical- or diet-induced models, and gene-engineered models have been developed and studied to elucidate molecular mechanisms and functional alterations associated with metabolic diseases. In this special issue, we aimed to provide information on recent developments on experimental animal models in this field and up-to-date information on the pathophysiology, therapeutic drugs/strategies, and diagnosis of metabolic diseases. Here, we bring together 6 excellent articles related to diabetes and related metabolic diseases from all over the world.

The review article “Focusing on Sodium Glucose Cotransporter-2 and the Sympathetic Nervous System: Potential Impact in Diabetic Retinopathy” by L. Y. Herat et al. comprehensively covers the etiology of diabetic retinopathy (DR) and highlights novel targets, SGLT2 and the sympathetic nervous system, for the management of this pathology. DR is a serious microvascular complication observed in many diabetic patients that can ultimately lead to vision loss. Prevention of DR and other diabetes-related microvascular complications is a major treatment goal. Today, SGLT2 inhibitors provide a new therapeutic option to control blood glucose levels and prevent the subsequent development of diabetic complications in retinal microvasculature. This in-time review summarizes current evidence on the use of SGLT2 inhibitors and identifies gaps that need to be addressed.

In the article entitled “Spontaneously Diabetic Torii (SDT) Fatty Rat, a Novel Animal Model of Type 2 Diabetes Mellitus, Shows Blunted Circadian Rhythms and Melatonin Secretion,” K. Sakimura et al. demonstrate the deficits in the circadian rhythms and dysregulation of melatonin secretion in SDT fatty rat, a new animal model of type 2 diabetic obesity. Since hyperglycemia, hyperlipidemia, and insulin resistance are all observed in these SDT fatty rats from a young age, this exciting new animal model will undoubtedly be useful for future studies investigating the relationship between deficits in the circadian rhythm and metabolic dysfunction in obese type 2 diabetics.

Nitric oxide (NO) is a potent vasodilator released from vascular endothelial cells and plays a crucial role in vascular homeostasis. In many cardiovascular diseases and type 2 diabetes, NO bioavailability is reduced primarily due to endothelial dysfunction. It is also known that metabolic syndrome including obesity and glucose tolerance is associated with impairment of NO signaling. Dietary supplementation of nitrate is an alternate source of NO when the endothelium-dependent, endogenous NO synthesis system is compromised. V. B. Matthews et al. examined whether the dietary supplementation of nitrate prevents the development of the metabolic syndrome in mice fed a high-fat diet, in their paper “Long-Term Dietary Nitrate Supplementation Does Not Prevent Development of the Metabolic Syndrome in Mice Fed a High-Fat Diet.” The authors also discuss the importance of short-chain fatty acids in the context of metabolic syndrome.

Abnormal platelet function such as platelet hyperreactivity and hyperaggregability is commonly observed in metabolic syndrome. It has been reported that arginine supplementation and aerobic exercise training enhance vascular NO activity, resulting in the inhibition of platelet hyperaggregability. However, the mechanisms underlying these beneficial effects remain unclear. In the article entitled “Aerobic Training Associated to Arginine Supplementation Reduces Platelet Hyperaggregability Collagen-Induced in Rats under High Risk to Develop Metabolic Syndrome,” Motta et al. examined the impact of aerobic training and/or arginine supplementation on platelet hyperaggregability, inflammatory mediators (i.e., IL-6 and IL-8), serum lipid profile, and serum lipid peroxidation in fructose-administered rats. The authors demonstrate that the combination of aerobic training and arginine supplementation provides benefit by prevention of collagen-induced platelet hyperaggregability and reduction of inflammatory markers that are not observed animal groups receiving either only aerobic training or only arginine supplementation. These findings suggest that the combination of currently available therapeutic options has greater benefit than monotherapy to delay the onset of cardiovascular diseases in patients with metabolic syndrome.

Vanadium derivatives have hypoglycemic effects in animal models and humans. Due to their role on insulin signaling and enzymatic processes regulation, these compounds are clinically used to patients with poorly controlled type 2 diabetes. “Vanadyl Sulfate Effects on Systemic Profiles of Metabolic Syndrome in Old Rats with Fructose-Induced Obesity” written by Ortega-Pacheco et al. describes the antiobese, hypoglycemic, and hypolipidemic effects of vanadyl sulfate on fructose-induced metabolic syndrome in aged rats. In addition to an antiobesity effect in aged obese rats, vanadyl sulfate improved insulin sensitivity and oral glucose tolerance tests in rats with fructose-induced chronic obesity. Vanadyl sulfate may be a valuable therapeutic agent in preventing insulin resistance, the development and progression of obesity, and metabolic syndrome complications in aged patients with obesity or type 2 diabetes.

N. Babaya et al. demonstrate that NSY-Chr14 is a streptozotocin- (STZ-) susceptible chromosome and that the STZ-susceptible region is located in the distal segment of NSY-Chr14 in their paper “Verification That Mouse Chromosome 14 Is Responsible for Susceptibility to Streptozotocin in NSY Mice.” Construction of new congenic strains will lead to fine mapping and identification of causal variants of the genes responsible for STZ susceptibility in the NSY mouse.

We hope these articles bring further light in the research field of diabetes and related metabolic diseases and contribute to the development of new therapeutic strategies and drugs in the future.

